# Resin-supported iridium complex for low-temperature vanillin hydrogenation using formic acid in water†

**DOI:** 10.1039/d1ra01460a

**Published:** 2021-04-28

**Authors:** Christene A. Smith, Francesco Brandi, Majd Al-Naji, Ryan Guterman

**Affiliations:** Department of Colloid Chemistry, Max Planck Institute of Colloids and Interfaces Am Mühlenberg 1 14476 Potsdam Germany Ryan.guterman@mpikg.mpg.de

## Abstract

Biorefinery seeks to utilize biomass waste streams as a source of chemical precursors with which to feed the chemical industry. This goal seeks to replace petroleum as the main feedstock, however this task requires the development of efficient catalysts capable of transforming substances derived from biomass into useful chemical products. In this study, we demonstrate that a highly-active iridium complex can be solid-supported and used as a low-temperature catalyst for both the decomposition of formic acid (FA) to produce hydrogen, and as a hydrogenation catalyst to produce vanillyl alcohol (VA) and 2-methoxy-4-methylphenol (MMP) from vanillin (V); a lignin-derived feedstock. These hydrogenation products are promising precursors for epoxy resins and thus demonstrate an approach for their production without the need for petroleum. In contrast to other catalysts that require temperatures exceeding 100 °C, here we accomplish this at a temperature of <50 °C in water under autogenous pressure. This approach provides an avenue towards biorefinery with lower energy demands, which is central to the decentralization and broad implementation. We found that the high activity of the iridium complex transfers to the solid-support and is capable of accelerating the rate determining step; the decomposition of FA into hydrogen and carbon dioxide. The yield of both VA and MMP can be independently tuned depending on the temperature. The simplicity of this approach expands the utility of molecular metal complexes and provides new catalyst opportunities in biorefinery.

## Abstract 2

Solid-supported molecular catalysis for biorefinery. Hydrogenation using formic acid in water at low temperature.

## Graphical abstract

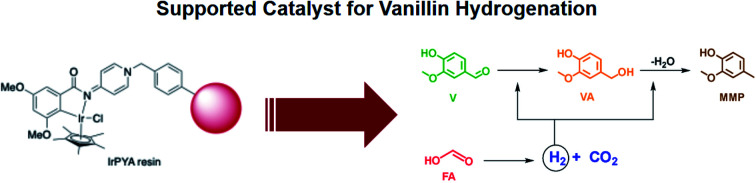

## Introduction

Catalysis is useful for a variety of transformations to synthesize pharmaceuticals and other value-added chemicals. Traditionally, metal complexes are used in catalysis because they are easily studied and modified to increase reactivity. One salient problem with them is their removal, which requires additional steps, solvent, and columns, nullifying the benefits of lower heat and time that they provide. Heterogeneous catalysts do not have the advantage of obtaining mechanistic insight and undergoing optimization to the degree of homogeneous catalysts, but they are easily removed from solution and do not require the intense work up procedures, unlike homogeneous catalysts. They also have the advantage of recyclability, which is crucial on an industrial scale for both environmental and economic reasons. Immobilization of known molecular catalysts onto a solid support harnesses the benefits of both homogeneous and heterogeneous catalysts with rational design and ease of removal. Recyclability with homogeneous catalysts is possible, but the process is often costly and time consuming. Immobilization of molecular catalysts with known high activity on solid supports makes their recyclability accessible in industry and is one strategy to overcome their troublesome recovery. The potential of catalysis is often limited by the scale up necessary for industrial application and this is particularly the case in biorefinery,^1–5^ which is an approach geared towards upgrading lignocellulosic biomass to valued-added chemicals in the so-called biorefinery processing.^3,6^ Within this process, lignocellulosic biomass undergoes physico-chemicals transformation toward low-carbon footprint fine products.^7^ Among these reactions, catalytic hydrogenation is a key reaction which allows the production of a wide range of compounds.^8–10^ Typically, a biomass heterogeneously-catalyzed hydrogenation reaction is conducted using relatively high temperature (100–200 °C), external pressurized H_2_ as reductive agent and supported metals as catalyst, such as Pt, Pd, Ru and Ni.^8,9,11–14^ Heterogeneous catalysis is the preferred method because it allows for the implementation of more economically efficient and environmentally benign reactor designs. However, biorefinery processes are expensive when compared to traditional refineries. In fact, the complexity of lignocellulosic biomasses requires a large number of cost-effective processing steps. Reducing the reaction temperature and pressure using new catalysts,^2^ replacing H_2_ with formic acid (FA),^14–18^ and achieving this using solid-supported catalysts are three viable strategies to reduce the cost of biorefinery. Innate difficulties in transporting and storing H_2_ poses a significant hurdle to biorefinery, where hydrogenation reactions are invariably needed to convert oxygen-rich biomaterial into useful substances for the chemical industry. Formic acid has emerged as a solution^19^ since it can be easily stored as a liquid and can be produced at low temperature from CO_2_ and H_2_ or by biomass processing.^20^ Furthermore, current efforts to efficiently produce formic acid seeks to bolster the “formic acid economy” as a pillar for future endeavours in green energy and biorefinery.^21^ Formic acid decomposition to hydrogen needs the assistance of a strong catalyst in order to lower the overall temperature of the reaction. Ru and Ir molecular catalysts are among the most commonly used for this purpose a wide variety of structures producing different dehydrogenation capabilities with dehydrogenation occurring at low temperature in many cases.^22^ Iridium is known to be active in this reaction as known by Himeda.^23^ It is also known to be an efficient hydrogenation catalyst of carbonyls as shown by Albrecht and co-workers.^24^ Therefore it is an ideal catalyst not just to convert formic acid to hydrogen, but to also convert a potential lignin feedstock to various other value-added chemicals at low temperature.

Vanillin (V) is nowadays the major lignin-derived feedstock already produced in industrial scale, mostly by Borregaard company.^25,26^ Vanillin itself is already used as a flavor in the food and cosmetic industry.^27^ Vanillin has the potential to be upgraded to value-added compounds *via* catalytic hydrogenation. Indeed, the hydrogenation products of vanillin, *i.e.* Vanillyl alcohol (VA) and 2-methoxy-4-methylphenol (MMP), can be used respectively, as flavor and bio-fuel, while both are promising precursors of sustainable epoxy resins.^8,28,29^

**Scheme 1:**
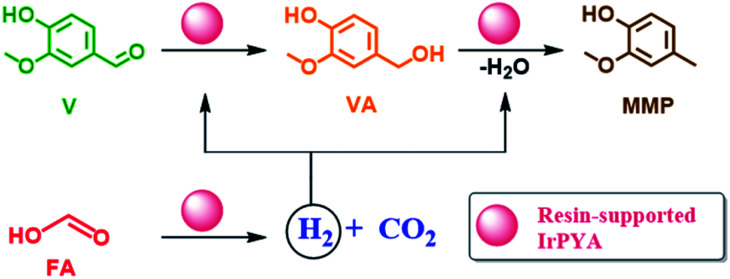
Hydrogenation of vanillin (V) to vanillyl alcohol (VA) and subsequently to 4-methyl-2-methoxyphenol (MMP) using formic acid as a reducing agent over IrPYA resin catalyst.

Vanillin hydrogenation proceeds toward VA and then presents a hydrodeoxygenation step to MMP, *viz.* in Scheme 1. To date, several studies have studied V hydrogenation, using supported metal catalysts (Pd, Ru, Rh, Pd, Au, Pt and Ni) under pressurized H_2_.^8,30–33^ For example, Bindwal *et al.* reported VA and MMP yield of 90 mol% and 10 mol% respectively, at 65 °C with Ru/C and 2.1 MPa of H_2_.^33^ Similarly, we recently reported on a complete conversion of V to MMP at 150 °C and 2.5 MPa using Ni supported on nitrogen doped carbon catalysts in a continuous flow system.^8^ As it can be seen, most reported studies of V hydrogenation reported a high V conversion and VA and MMP yields but at relatively high temperatures (60–150 °C) and H_2_ pressure (2–2.5 MPa). However, the utilization of alternative and sustainable hydrogen sources *e.g.*, FA, and the performance of V hydrogenation at room temperature was scarcely reported.^14,34,35^ For biorefinery in particular, low-temperature and low-pressure methods offer reduced net energy demand, more simplistic setups and finally the need to centralize the biorefinery process in order to make the heat requirements economically feasible. At the moment, biorefinery centralization is required in order to reduce net energy demands by recycling heat, however low-temperature approaches allows for decentralization and thus reduces transportation costs for raw materials. For example, a study examining the cost of straw valorization in Baden-Wuerttemberg, Germany, found that a centralized biorefinery approach results in a transportation cost of 10% of the total cost for straw.^36^ Furthermore, they explained that smaller, decentralized biorefineries offer better price-to-quantity ratios and are less sensitive to feedstock cost fluctuations. This is in contrast to larger biorefineries that require a constant supply of larger volumes of low-cost material and thus become less profitable when feedstock value increase in price. For these reasons, efforts towards smaller, decentralized biorefineries *via* lower heating requirements and simpler setups can improve the profitability of biorefinery.

In this context, we report the use of a solid-supported iridium complex as a low-temperature catalyst for V upgrading using FA. Utilizing a molecular complex provides the benefit of harnessing developed methodologies for homogenous catalysts, but with the convenience of a heterogeneous catalyst for separation. The resin supported Ir catalyst (IrPYA) was able to decompose FA into hydrogen at low temperature (50 °C), with maximum FA conversion of 76 mol%. The supported iridium catalyst was also found to be active for V hydrogenation toward VA and MMP in the presence of FA as a hydrogen source. Product selectivity was found tunable with temperature, yielding a maximum of 54 mol% of VA at 25 °C and a maximum of 24 mol% of MMP at 50 °C. These results demonstrate how metal complexes may be harnessed on solid-supports and their use expanded in low-temperature and low-pressure biorefinery.

## Results and discussion

### IrPYA resin synthesis

**Scheme 2:**
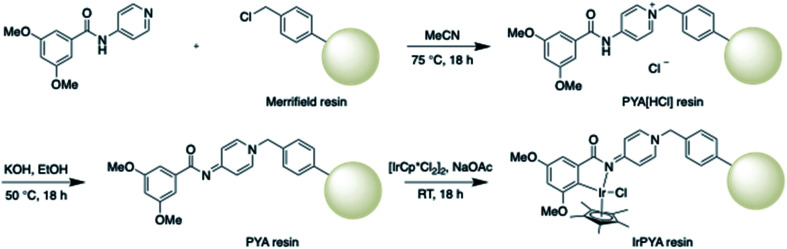
Synthesis of the IrPYA resin.

**Fig. 1:**
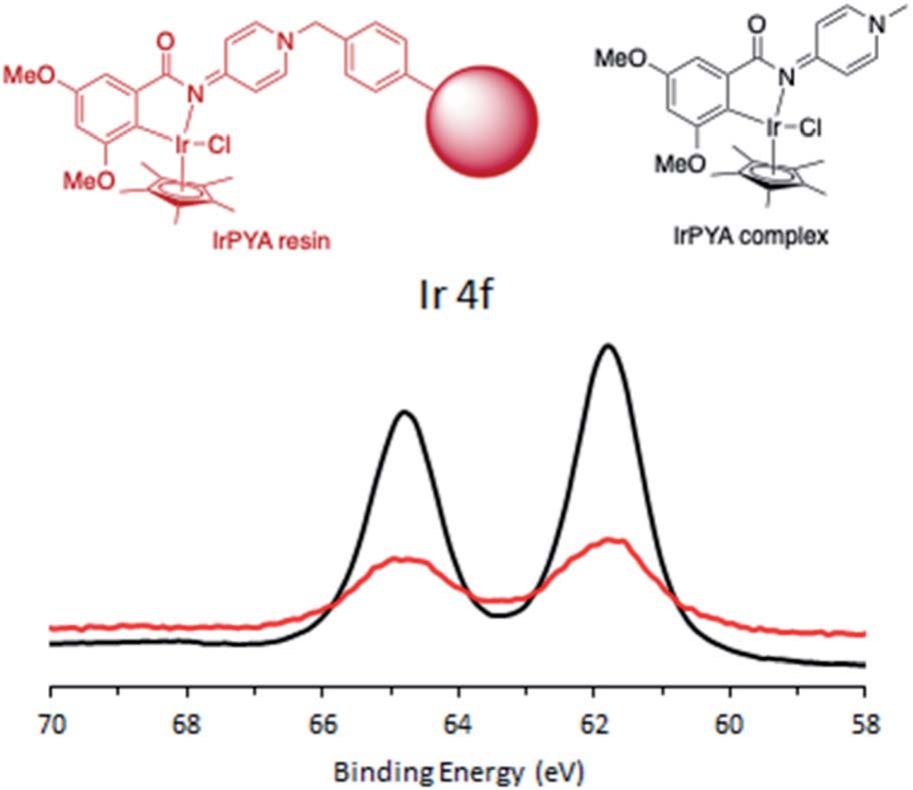
Ir 4f region of the XPS spectra of the IrPYA resin (red) and the IrPYA complex (black).

First 3,5-dimethoxy-*N*-(pyridin-4-yl)benzamide was reacted with Merrifield resin in acetonitrile at 75 °C for 18 hours (Scheme 2). After the reaction, the resin was washed several times with hot acetonitrile to remove any precursor and then dried to give PYA[HCl] resin. Reaction completion was observed by FTIR (Fig. S1 in the ESI†). Then an altered procedure for proton removal was done by reacting the PYA[HCl] resin in a solution of KOH in EtOH at 50 °C overnight to give the PYA resin (Scheme 2). The resin was washed with hot EtOH and then H_2_O to remove any KOH. The material was then washed with acetone to remove any H_2_O and then dried in a vacuum oven. The prepared ligand resin (PYA resin) was observed by FTIR (Fig. S2 in the ESI†). Then metalation of the supported ligand was done by reacting the PYA resin with [IrCp*Cl_2_]_2_ and NaOAc in dichloromethane at room temperature for 18 hours (Scheme 2). The material was then washed with dichloromethane, H_2_O, and acetone before drying. The final supported catalyst material IrPYA resin was confirmed by FTIR (Fig. S3 in the ESI†). In all cases FTIR was compared with a molecular species for confirmation of reaction completion (Fig. S1–S3 in the ESI†) and in the case of the metalation reaction XPS analysis was also performed on both the molecular and supported catalyst for additional confirmation (Fig. 1).

The XPS spectra of the IrPYA resin material matched with that of the homogeneous IrPYA complex, showing a single oxidation state (Fig. 1). The IrPYA resin has a 4f_5/2_ peak at 64.8 eV and the 4f_7/2_ peak at 61.8 eV (red, Fig. 1), and the IrPYA complex shows a 4f_5/2_ peak at 64.8 eV and a 4f_7/2_ peak at 61.7 eV (black, Fig. 1). This shows that the synthesized material contains a molecular catalyst and no nanoparticle formation is observed on the surface of the resin.

### Catalyst evaluation

**Fig. 2:**
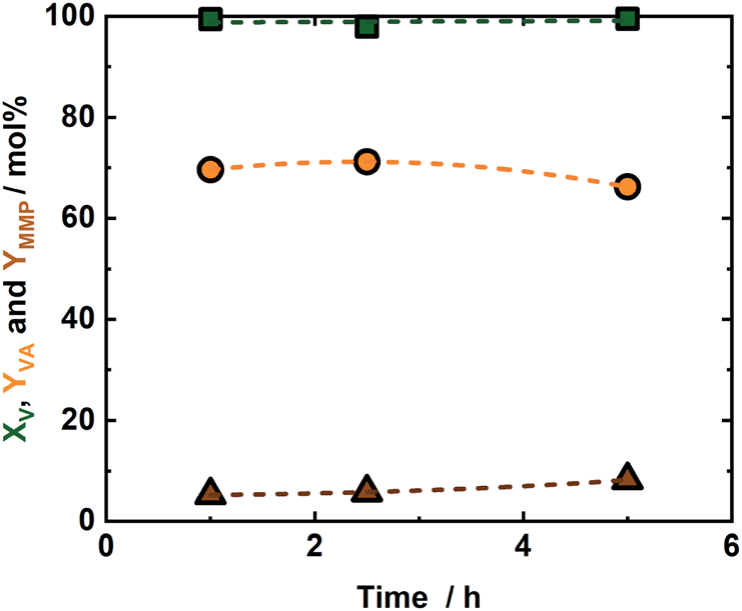
The conversion of vanillin (*X*_V_) and the yield of vanillyl alcohol and 4-methyl-2-methoxyphenol (*Y*_VA_ and *Y*_MMP_) as a function of the reaction time; reaction conditions: *c*_V_ = (715 μL, 1 wt%, 0.047 mmol), *c*_FA_ = (293 μL, 7.78 mmol), *c*_KOH_ (515 μL, 2 M, 1.03 mmol), *T* = 25 °C and autogenous pressure (<1.0 MPa).

An iridium complex was successfully supported on Merrifield resin and used as a catalyst for the simultaneous decomposition of FA and production of VA and MMP from V. The activity of the synthesized IrPYA resin catalyst in the hydrogenation of V to VA and the subsequent product MMP was studied using FA as a reducing agent in the presence of KOH (Scheme 1). The performance of the catalyst was initially investigated at room temperature (25 °C), with samples collected at different reaction times (after 1 h, 2.5 h and 5 h), *viz.* in Fig. 2. After 1 h, the conversion of V was 99 mol% with a VA and MMP yield of 69 mol% and 5.2 mol% respectively. This demonstrates that a single catalyst can both dehydrogenate FA and hydrogenate V and VA within a reaction vessel. The solution after the reaction remained colourless, indicating that the strongly coloured metal center remained bound to the resin. Increasing the reaction time from 1 h to 2.5 h did not result in a significant increase of *Y*_VA_ (from 69 mol% to 70 mol%) and *Y*_MMP_ (from 5.2 mol% to 5.8 mol%), *cf.*Fig. 2. To correlate catalyst performance in V hydrogenation to FA decomposition towards H_2_ and CO_2_, a separate experiment was conducted to investigate the FA degradation rate in absence of V, *cf.* Fig. S6 at ESI.† In this case, FA conversion, increases from 6.7 mol% to 15.7 mol% after 2.5 h, *cf.* Fig. S6 in ESI.† These results combined with the insignificant change in V conversion between 1 and 2.5 h indicates that the Ir active sites are occupied firstly by FA. A further extension of the reaction time to 5 h resulted in an increase of *Y*_MMP_ from 5.8 mol% to 8.2 mol% combined with reduction of *Y*_VA_ from 71 mol% to 66 mol% as a result of its conversion to MMP. This increase of MMP yield is correlated to the increase in FA conversion from 15.7 mol% to 21 mol%, which liberated the active Ir sites to V that undergoes to hydrogenation reaction (Fig. S6†). This observation indicates that the decomposition of FA to H_2_ and CO_2_ is the rate determining step for the hydrogenation of VA to MMP and is in-line with our previous studies for hydrogenation of levulinic acid and V as a model mixture,^14^ as well as γ-valerolactone hydrogenation to pentanoic acid using formic acid as a reducing agent.^14^ Moreover, the 21 mol% conversion of FA at room temperature is a comparable value to similar studies conducted with homogeneous Ir-catalyst,^37^ but here obtained with immobilized IrPYA which can potentially open new application in the field of heterogeneous catalysis and fuel cells.^38–40^ As this catalyst uniquely contains the IrPYA supported on Merrifield resin (the catalyst is in solid form) and the IrPYA not used as homogenous catalyst, as well as the reaction conditions is mild (*T* = 50 °C and autogenous pressure), we anticipate that no difference in the catalytic performance should be observed. In addition, research on this issue coupled with advanced characterization techniques are ongoing at our laboratory to further understand the unique performance and its potential scalability.

**Fig. 3:**
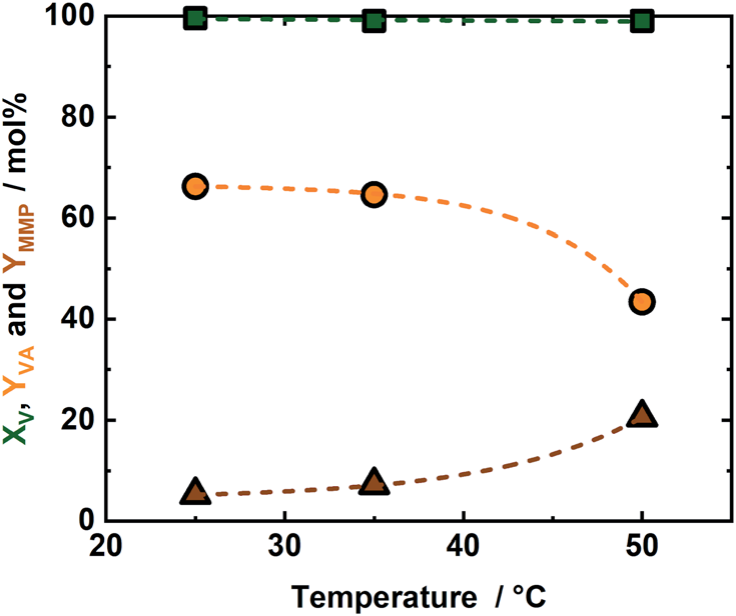
The conversion of vanillin (*X*_V_) and the yield of vanillyl alcohol and 4-methyl-2-methoxyphenol (*Y*_VA_ and *Y*_MMP_) as a function of the reaction temperature; reaction conditions: *c*_V_ = (715 μL, 1 wt%, 0.047 mmol), *c*_FA_ = (293 μL, 7.78 mmol), *c*_KOH_ (515 μL, 2 M, 1.03 mmol), reaction = 5 h and autogenous pressure (<1.0 MPa).

In order to maximize the *Y*_MMP_ the reaction temperature was increased from 25 °C to 35 °C and subsequently to 50 °C (Fig. S6–S8 in the ESI†). The experiment conducted at 35 °C resulted in only slight improvement of *Y*_MMP_ when compared to the 25 °C experiment (Fig. 3 and S9 in the ESI†). A further increase in reaction temperature to 50 °C, reduced *Y*_VA_ from 54 mol% to 44 mol% and increased *Y*_MMP_ from 8 mol% to 24 mol%, *cf.*Fig. 3 and S8 in the ESI.† This high *Y*_MMP_ is correlated to the high *X*_FA_ (76 mol%) at 50 °C after 5 h of reaction time (Fig. 3), which leads to a higher availability of active sites with respect to the experiments at 25 °C and 35 °C. Finally and noteworthy to be mentioned, the mass balance loss of ∼23 mol% is attributed to deposition of the VA and MMP on the surface of the catalyst due to its low solubility in water.^41^ This *Y*_MMP_ is relatively low if compared to a similar study conducted at same temperature with pressurized H_2_ as the hydrogen source or with FA as the hydrogen source but at higher temperature, but is based using milder condition.^14,35^ Moreover, the selectivity toward VA or MMP can be tuned according to temperature.

**Fig. 4:**
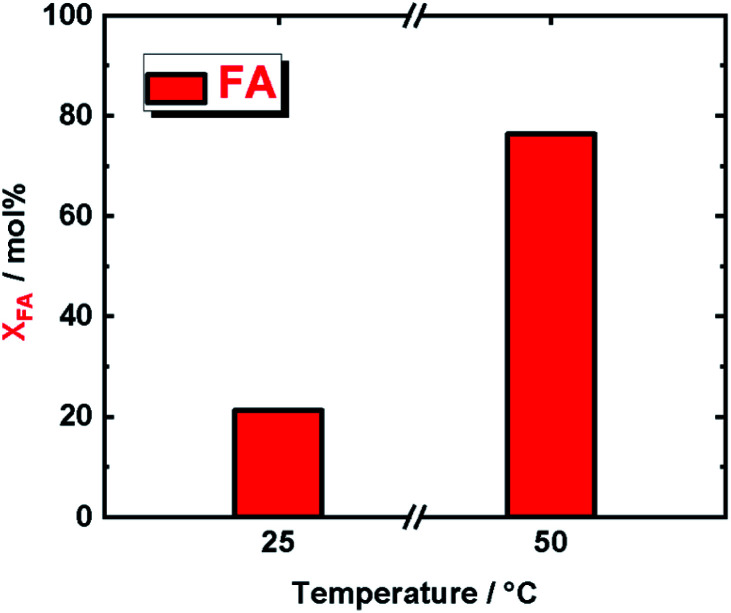
A comparison in the conversion of formic acid (*X*_FA_) at 25 °C and 50 °C after 5 h of reaction time; reaction conditions; *c*_FA_ = (293 μL, 7.78 mmol), *c*_KOH_ (515 μL, 2 M, 1.03 mmol), and autogenous pressure (<1.0 MPa).

In order to confirm that our supported molecular catalyst, IrPYA resin, was indeed decomposing formic acid we subjected it to the same reaction conditions as in the V to VA and MMP reactions at both 25 °C and 50 °C (Fig. 4). At 25 °C we observed a decomposition *X*_FA_ of 21% and at 50 °C a *X*_FA_ of 76%. Although the conversion of formic acid at room temperature is approximately a third of its conversion at 50 °C, this still provides an excess of hydrogen for the conversion of V to VA and MMP in our reaction, as we used a large excess. Moreover, the FA decomposition at this lower temperatures, *i.e.* 25 °C and 50 °C, is typical of Ir complexes used as homogeneous catalysts.^22^ This finding confirm that immobilized IrPYA has the same catalytic activity as homogenous Ir catalysts. Extending these findings in biorefinery will require flow reactors to be designed that take advantage of the dual behaviour of IrPYA supported on Merrifield resin. Using this catalyst in our previously reported continuous flow system for hydrogenation of vanillin will result in lower reaction temperature (from 150 °C to 50 °C), as well as a reduction in system pressure from 6.0 MPa to below 1.0 MPa. All these will lead to efficient and selective process coupled with high economic efficiency biorefinery process (due to mild operating conditions). In addition, this type of hydrogenation with IrPYA supported on Merrifield resin can be expanded to wide range of biomass-derived compounds such as glucose, 5-hydroxymethylfurfural, levulinic acid and γ-valerolactone.

## Conclusions

An iridium complex was successfully supported on Merrifield resin and used as a catalyst for the simultaneous decomposition of FA and production of VA and MMP from V. This approach harnesses the high activity and tunability of molecular metal complexes, but with the convenience and ease of separation of heterogeneous catalysts. Catalytic production of MMP typically takes place at temperatures exceeding 100 °C, however here it was accomplished at temperatures <50 °C. This was achieved by taking advantage of the high activity of the iridium complex, which is capable of accelerating the rate determining step; the decomposition of FA to H_2_ and CO_2_. Once this occurs, hydrogenation of V and VA proceeds using the same iridium complex as an active hydrogenation catalyst. As well, the VA and MMP yield was found to be tunable with temperature, providing a convenient means to tune product formation. While iridium is an expensive metal, it promotes mild reaction conditions and thus helps to offset other costs including heating and equipment designed for high temperature and pressures. As well, we believe that as formic acid becomes more integrated into the green economy, its use in biorefinery as a hydrogen source will become more prevalent. These results provide new prospects for decentralized biorefinery *via* low energy demands and expands the Frontier for supported molecular metal complexes in biomass upgrading.

## Author contributions

Christene A. Smith and Francesco Brandi were the prime experimentalists. Ryan Guterman and Majd Al-Naji supervised the research.

## Conflicts of interest

There are no conflicts to declare.

## Supplementary Material

RA-011-D1RA01460A-s001

## References

[cit1] Corma Canos A., Iborra S., Velty A. (2007). Chem. Rev..

[cit2] Anastas P., Eghbali N. (2010). Chem. Soc. Rev..

[cit3] Esposito D., Antonietti M. (2015). Chem. Soc. Rev..

[cit4] Guterman R., Molinari V., Josef E. (2019). Angew. Chem..

[cit5] Mendoza Mesa J. A., Brandi F., Shekova I., Antonietti M., Al-Naji M. (2020). Green Chem..

[cit6] Al-Naji M., Schlaad H., Antonietti M. (2020). Macromol. Rapid Commun..

[cit7] Liao Y., Koelewijn S. F., van den Bossche G., van Aelst J., van den Bosch S., Renders T., Navare K., Nicolaï T., van Aelst K., Maesen M., Matsushima H., Thevelein J. M., van Acker K., Lagrain B., Verboekend D., Sels B. F. (2020). Science.

[cit8] Brandi F., Bäumel M., Molinari V., Shekova I., Lauermann I., Heil T., Antonietti M., Al-Naji M. (2020). Green Chem..

[cit9] Besson M., Gallezot P., Pinel C. (2014). Chem. Rev..

[cit10] Climent M. J., Corma A., Iborra S. (2014). Green Chem..

[cit11] Brandi F., Bäumel M., Shekova I., Molinari V., Al-Naji M. (2020). Sustainable Chem..

[cit12] Al-Naji M., Balu A. M., Roibu A., Goepel M., Einicke W. D., Luque R., Gläser R. (2015). Catal. Sci. Technol..

[cit13] Bailey S. and King F., in Fine Chemicals through Heterogeneous Catalysis, Wiley-VCH Verlag GmbH, Weinheim, Germany, 2007, pp. 351–471

[cit14] Al-Naji M., Popova M., Chen Z., Wilde N., Gläser R. (2020). ACS Sustainable Chem. Eng..

[cit15] Al-Naji M., Van Aelst J., Liao Y., D'Hullian M., Tian Z., Wang C., Gläser R., Sels B. F. (2020). Green Chem..

[cit16] Gilkey M. J., Xu B. (2016). ACS Catal..

[cit17] De S., Saha B., Luque R. (2015). Bioresour. Technol..

[cit18] Al-Naji M., Yepez A., Balu A. M., Romero A. A., Chen Z., Wilde N., Li H., Shih K., Gläser R., Luqueb R. (2016). J. Mol. Catal. A: Chem..

[cit19] van Putten R., Wissink T., Swinkels T., Pidko E. A. (2019). Int. J. Hydrogen Energy.

[cit20] Nielsen M., Alberico E., Baumann W., Drexler H. J., Junge H., Gladiali S., Beller M. (2013). Nature.

[cit21] Weilhard A., Argent S. P., Sans V. (2021). Nat. Commun..

[cit22] Barnard J. H., Wang C., Berry N. G., Xiao J. (2013). Chem. Sci..

[cit23] Himeda Y. (2009). Green Chem..

[cit24] Navarro M., Smith C. A., Albrecht M. (2017). Inorg. Chem..

[cit25] Fache M., Boutevin B., Caillol S. (2016). ACS Sustainable Chem. Eng..

[cit26] Borregaard, Smart Vanillin

[cit27] Ilic I. K., Meurer M., Chaleawlert-Umpon S., Antonietti M., Liedel C. (2019). RSC Adv..

[cit28] Liguori F., Moreno-Marrodan C., Barbaro P. (2020). Chem. Soc. Rev..

[cit29] Meylemans H. A., Groshens T. J., Harvey B. G. (2012). ChemSusChem.

[cit30] Nie R., Yang H., Zhang H., Yu X., Lu X., Zhou D., Xia Q. (2017). Green Chem..

[cit31] Shit S. C., Singuru R., Pollastri S., Joseph B., Rao B. S., Lingaiah N., Mondal J. (2018). Catal. Sci. Technol..

[cit32] Santos J. L., Alda-Onggar M., Fedorov V., Peurla M., Eränen K., Mäki-Arvela P., Centeno M., Murzin D. Y. (2018). Appl. Catal., A.

[cit33] Bindwal A. B., Vaidya P. D. (2014). Energy Fuels.

[cit34] Pongthawornsakun B., Kaewsuanjik P., Kittipreechakun P., Ratova M., Kelly P., Mekasuwandumrong O., Praserthdam P., Panpranot J. (2020). Catal. Today.

[cit35] Hao P., Schwartz D. K., Medlin J. W. (2018). Appl. Catal., A.

[cit36] Petig E., Rudi A., Angenendt E., Schultmann F., Bahrs E. (2019). GCB Bioenergy.

[cit37] Lu S. M., Wang Z., Wang J., Li J., Li C. (2018). Green Chem..

[cit38] Guan C., Pan Y., Zhang T., Ajitha M. J., Huang K. (2020). Chem.–Asian J..

[cit39] Maenaka Y., Suenobu T., Fukuzumi S. (2012). Energy Environ. Sci..

[cit40] Eppinger J., Huang K. W. (2017). ACS Energy Lett..

[cit41] Yang X., Liang Y., Zhao X., Song Y., Hu L., Wang X., Wang Z., Qiu J. (2014). RSC Adv..

