# Supplementary material for: Resin-supported iridium complex for low-temperature vanillin hydrogenation using formic acid in water
Source: RSC Adv. 2021 Apr 28;11(26):15835–40. doi: 10.1039/d1ra01460a (PMC9030846; doi:10.1039/d1ra01460a)
Supplement: RA-011-D1RA01460A-s001 [file RA-011-D1RA01460A-s001.pdf]

Electronic Supplementary Material (ESI) for RSC Advances.
This journal is © The Royal Society of Chemistry 2021

SUPPORTING INFORMATION

Resin-supported iridium complex for low-temperature vanillin hydrogenation using formic acid in water

Christene A. Smith, Francesco Brandi, Majd Al-Naji, Ryan Guterman**

Contains 11 pages; Experimental-Characterization Details, and 11 Supporting Figures.

Materials

All materials were used as purchased unless stated otherwise. Dichloromethane (≥99.9%), Merrifield resin, and vanillin (>99%) were purchased from Sigma Aldrich. Acetonitrile (≥99.9%) was purchased from Merck Millipore. Vanillyl Alcohol (>99%) and 2-Methoxy-4-methoxyphenol (>99%) were provided by Acros Organic. Potassium hydroxide (≥), ethanol (≥), $[IrCp^*Cl_2]_2$ (≥), sodium acetate (≥), formic acid (≥), , DMSO-d_6 with 0.03 % TMS (≥). 3,5-dimethoxy-N-(pyridin-4-yl)benzamide, 4-(3,5-dimethoxybenzamido)-1-methylpyridin-1-ium chloride, 3,5-dimethoxy-N-(1-methylpyridin-4(1H)-ylidene)benzamide, and were prepared following the procedure from Albrecht and coworkers.[1]

Instrumentation

^1H and $^{13}C\{^1H\}$ NMR spectra were recorded on either a Bruker Avance 400 MHz or a Varian 400 MHz spectrometer. Chemical shifts are reported in delta (δ) units, expressed in parts per million (ppm) downfield from tetramethylsilane using residual protonated solvents as internal standards (^1H NMR DMSO-d_6: 2.50 ppm; $^{13}C\{^1H\}$ NMR DMSO- d_6: 39.52 ppm). All data were acquired using Bruker Topspin or Vnmr with a standard pulse-sequence library and were processed using MestReNova software. All measurements were carried out at 298 K.

TGA experiments were performed using the TG 209 F1 Libra from Netzsch with Al crucibles. A constant heating rate of 10.0 K/ min was used with N_2 gas purging. The amount of samples used for TGA was between 9 and 11 mg.

X-ray photoelectron spectroscopy (XPS) analysis was acquired using a Thermo Scientific K-Alpha+ X-ray photoelectron spectrometer. All samples were analysed using a microfocused, monochromated AlKα X-ray source (1486.68 eV; 400μm spot size). To prevent any localized charge build-up during analysis the K-Alpha+ charge compensation system was employed. Samples were mounted on conductive carbon tape. The signals of iridium, carbon, nitrogen and oxygen were scanned at a pass energy of 50 eV for high resolution spectra and 200 eV for survey spectra. Curve fitting of XPS C 1s, N 1s and Ir 4f core levels were fitted to Lorentzian line-shapes and Tougaard background fitting using CasaXPS software (v.2.3.22PR1.0). Binding energies were calibrated by taking the adventitious C 1s binding energy (284.8 eV) as a reference.

Inductively coupled plasma (ICP) measurements were performed using an Optima 8000 optical emission spectrometer from PerkinElmer.

Infrared (IR) measurements were performed using Nicolet iS5 fourier transform-infrared (FT-IR) spectrometer from Thermo Scientific.

Experimental

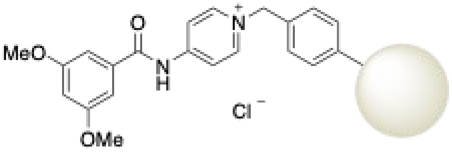

PYA[HCl] Resin

3,5-dimethoxy-*N*-(pyridine-4-yl)benzamide (2.000 g, mmol, eq), Merrifield resin (2.000 g), and acetonitrile (100 mL) were added to a pressure tube. The pressure tube was sealed and placed in a 75 °C oil bath for 18 hours. The vessel was then cooled to room temperature and the resin was filtered and washed three times with hot acetonitrile (3 x 50 mL). The acetonitrile washes were concentrated to recover the starting material to be used in subsequent reactions. The resin was then dried in a vacuum oven overnight and then analyzed by FTIR (**Figure S1**).

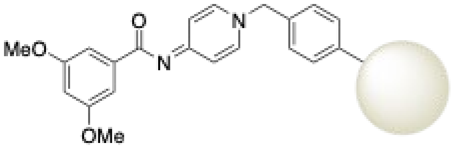

PYA Resin

The pyridinium salt resin (**PYA[HCl] Resin**, 1.000 g) was then placed in a round bottom flask in a solution of KOH in ethanol (1 M, 10 mL). The flask was then heated at 50 °C overnight. The resin was then filtered and washed with additional ethanol to remove any adsorbed salts. The resin was then dried in a vacuum oven overnight and then analyzed by FTIR (**Figure S2**).

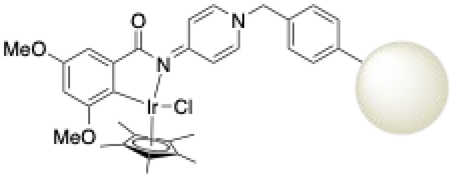

IrPYA Resin

The pyridylidene amide resin (**PYA Resin**, 1.500 g), [IrCp*Cl$_2$]$_2$ (0.9416 g, 1.18 mmol), and NaOAc (0.0968 g, 1.18 mmol) were dissolved in dry CH_2Cl_2 (50 mL) and stirred at room temperature for 18 h. After the reaction was completed the resin was washed with additional CH_2Cl_2 and H_2O to remove any salts from

the resin. The resulting red resin was then dried in a vacuum oven overnight and then analyzed by FTIR (**Figure S3**), XPS (**Figure S4-Figure S5**), and ICP (12.7 +/- 0.09 mg/g Ir determined by ICP).

Vanillin Hydrogenation experiment

5 mL of formic acid (293 uL, 7.78 mmol), vanillin (715 µL, 1 wt %, 0.047 mmol), and KOH (515 µL, 2 M, 1.03 mmol) were added to a pressure tube (from Ace glass incorporated USA type #7 model 8648-34, pressure rating of 1 MPa at 120°C). Then a sample was taken to account for T = 0. After this 50 mg of the **IrPYA Resin** was added and the pressure tube was sealed. The reaction was carried with the autogenous pressure generated by formic acid decomposition. The reaction was then carried out in duplicate at 25 °C, 35 °C, and 50 °C. For HPLC experiments samples of 50 µL were collected and diluted with 500 µL of H_2O before analysis to avoid saturation of the DAD by a highly concentrated solution. For NMR experiments samples of 10 µL were collected and measured with 500 µL of DMSO with 0.03 % TMS as an internal standard.

Vanillin Hydrogenation - Product analysis and quantification

Reactants and products of the Vanillin hydrogenation reactions were analysed using HPLC Agilent 1200 series equipped with a quaternary pump, a C18 column (Hypersil GOLD C18 Selectivity LC Series) column and with a diode array detector (DAD). The DAD detector was used to ensure a complete separation of the reactant and products peaks due to the presence of characteristic chromophores. All measurements were conducted using a wavelength of 280 nm. The HPLC program included an injection volume of 3.0 µL at 35° C. The total acquiring time was of 15 min with an eluent flow of 1.2 mL min^{-1}. The eluent phase consisting of an isocratic mixture of acetonitrile and water (50:50 vol.-%) has been used in the first 8 minutes. Then, the column was washed with a non-isocratic solution of 100 % acetonitrile between the 8th and 12th minutes to ensure the complete removal of compounds. Finally, the eluent composition has been reset to the initial isocratic phase. The products were identified by comparing the retention time with standard substances, therefore the retention time for the vanillin (**V**), vanillyl alcohol (**VA**) and 4-methyl,2-methoxyphenol (**MMP**) was 3.0 min, 2.7 min and 3.8 min, respectively.

The molar concentration (C_i) of the reactant and products was calculated from the integrated area of peaks and referred to the calibration curve conducted with standard compounds. The conversion and yield of the compounds (X_V, and $Y_{VA,MMP}$) were calculated as reported in equation **1** and **2**

$$\boldsymbol{X_V} = \frac{\boldsymbol{C_V^0} - \boldsymbol{C_V^T}}{\boldsymbol{C_V^0}} \cdot 100\% \quad \text{Eq.1}$$

$$\boldsymbol{Y_{VA,MMP}} = \frac{\boldsymbol{C_{VA,\,MMP}^T}}{\boldsymbol{C_V^0}} \cdot 100\% \quad \text{Eq.2}$$

where c^0_i and c^t_i indicates respectively the initial *i*-th compound concentration and the *i*-th compound concentration at the *T* distinct time.

Formic acid decomposition experiment

To a 5 mL pressure tube formic acid (293 µL, 7.78 mmol), KOH (515 µL, 2 M, 1.03 mmol), and H_2O (715 µL, 39.69 mmol) were added. Then a sample was taken to account for T = 0. After this 50 mg of the **IrPYA Resin** was added and the vessel was sealed. The reaction was carried out in duplicate at 25 °C and 50 °C. For NMR experiments samples of 10 µL were collected and measured with 500 µL of DMSO with 0.03 % TMS as an internal standard.

FTIR

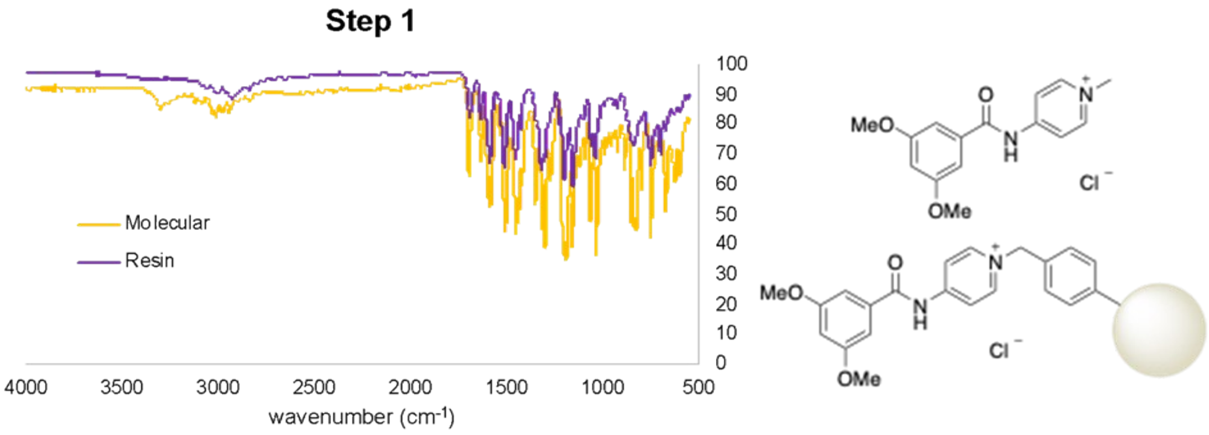

Figure S1: FTIR spectra of **PYA[HCl] Resin** (purple) and the molecular analog (yellow).

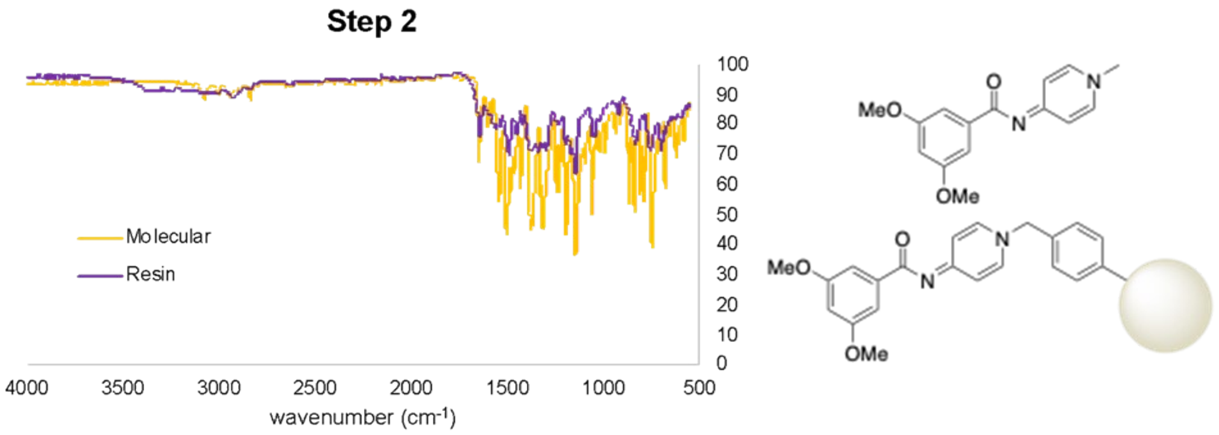

Figure S2: FTIR spectra of **PYA Resin** (purple) and the molecular analog (yellow).

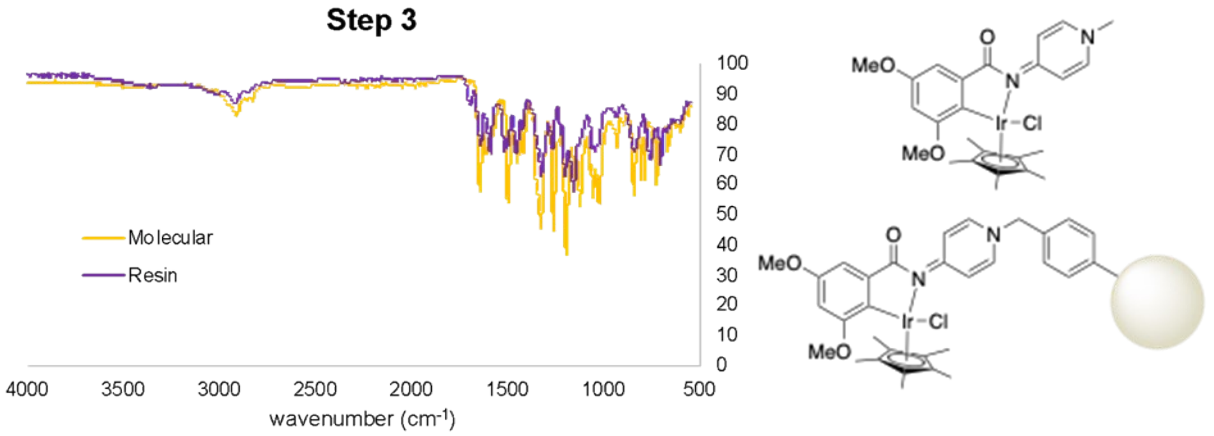

Figure S3: FTIR spectra of the **IrPYA Resin** (purple) and the molecular analog (yellow).

XPS

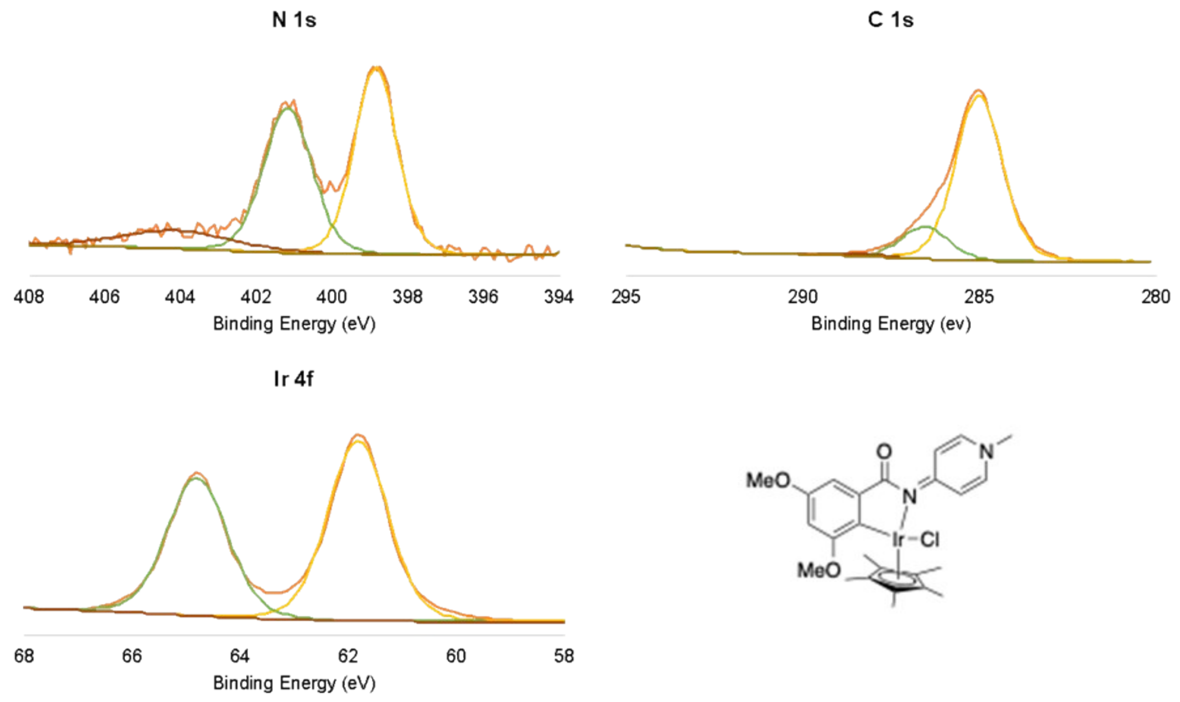

Figure S4: N 1s, C 1s, and Ir 4f XPS spectra of the molecular **IrPYA complex**.

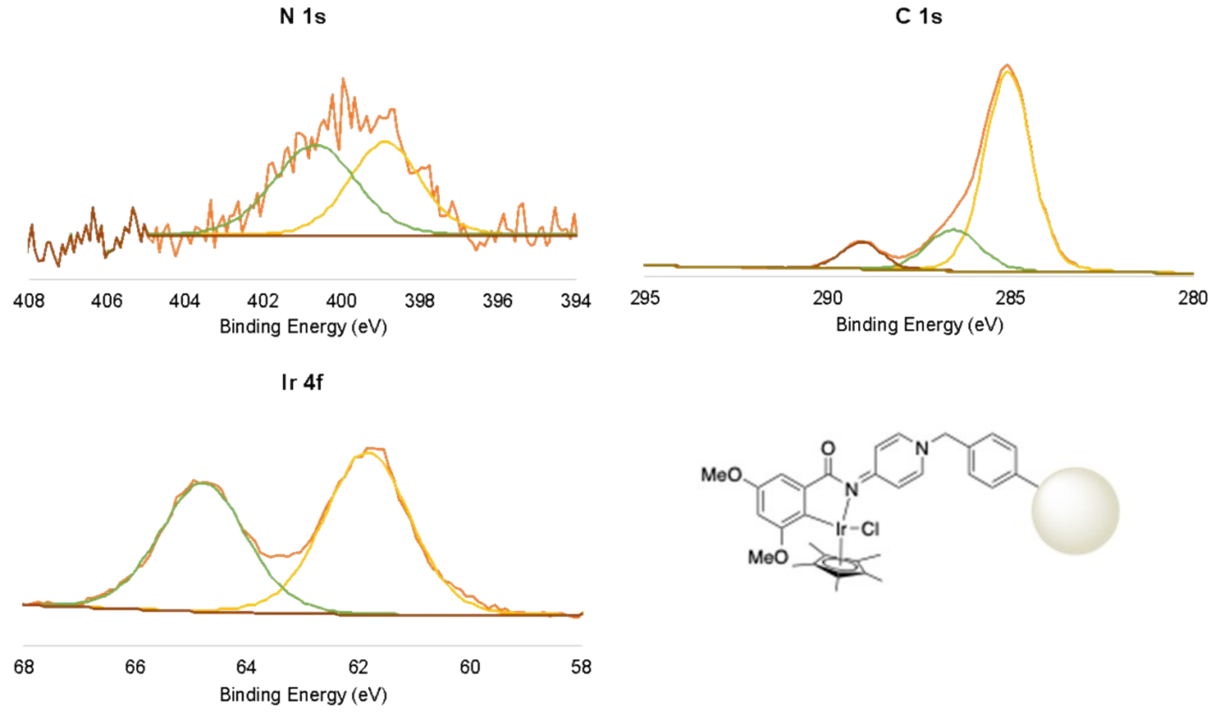

Figure S5: N 1s, C 1s, and Ir 4f XPS spectra of the **IrPYA Resin**.

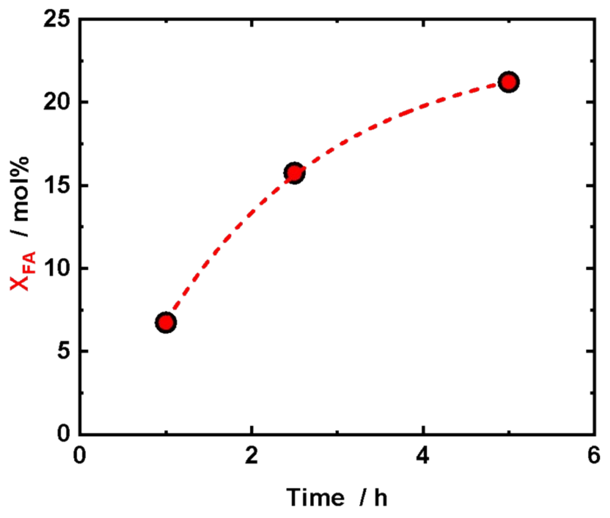

Figure S6: The conversion of formic acid (X_{FA}) as a function of the reaction time; Reaction conditions:, c_{FA} = 293 uL, 7.78 mmol), c_{KOH} (515 µL, 2 M, 1.03 mmol), T = 25◦C and autogenous pressure (<1.0 MPa).

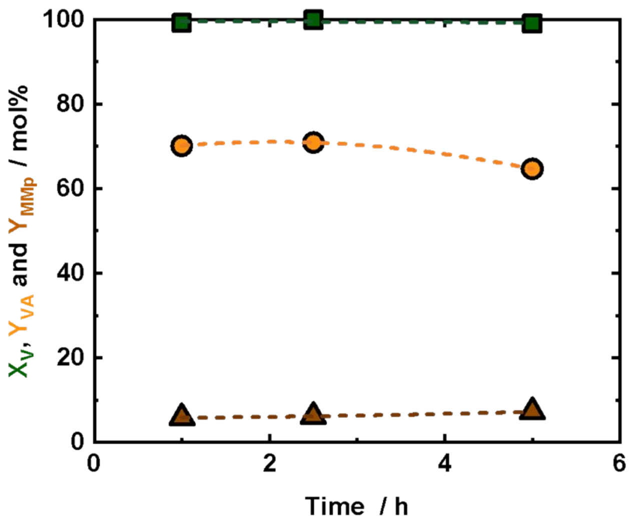

Figure S7: The conversion of vanillin (X_V) and the yield of vanillyl alcohol and 4-methyl-2-methoxyphenol ($Y_{VA\ and}$ Y_{MMP}) as a function of the reaction time; Reaction conditions: c_V = (715 µL, 1 wt %, 0.047 mmol), c_{FA} = 293 uL, 7.78 mmol), c_{KOH} (515 µL, 2 M, 1.03 mmol), T = 35◦C and autogenous pressure (<1.0 MPa).

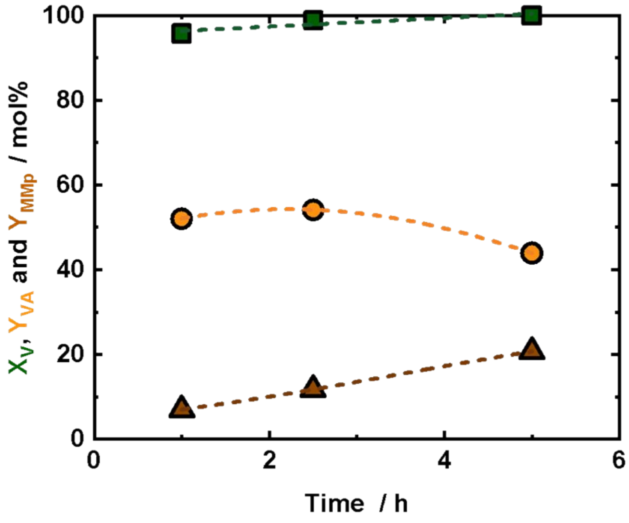

Figure S8: The conversion of vanillin (X_V) and the yield of vanillyl alcohol and 4-methyl-2-methoxyphenol (Y_{VA} and Y_{MMP}) as a function of the reaction time; Reaction conditions: c_V = (715 µL, 1 wt %, 0.047 mmol), c_{FA} = 293 uL, 7.78 mmol), c_{KOH} (515 µL, 2 M, 1.03 mmol), T = 50◦C and autogenous pressure (<1.0 MPa).

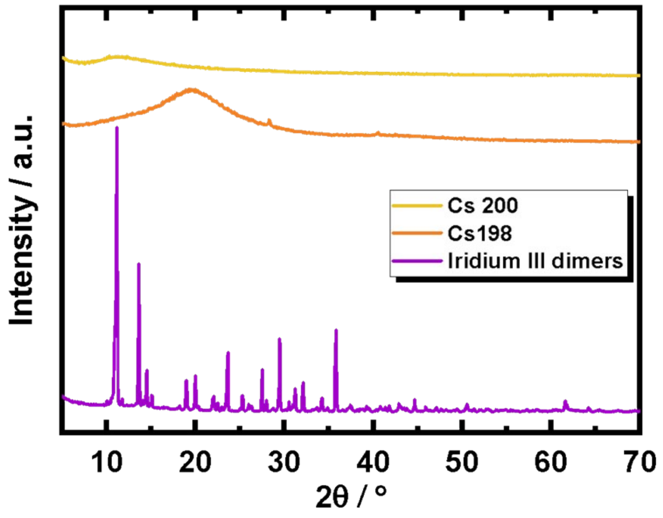

Figure S9: XRD patterns for Ir precursor.

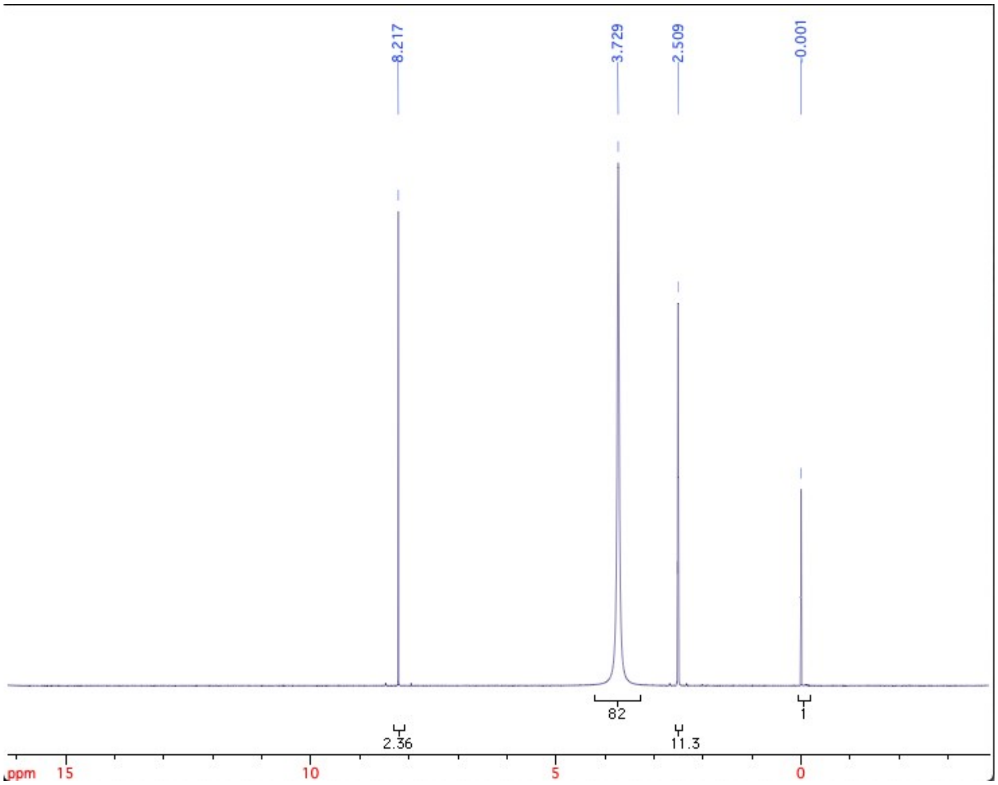

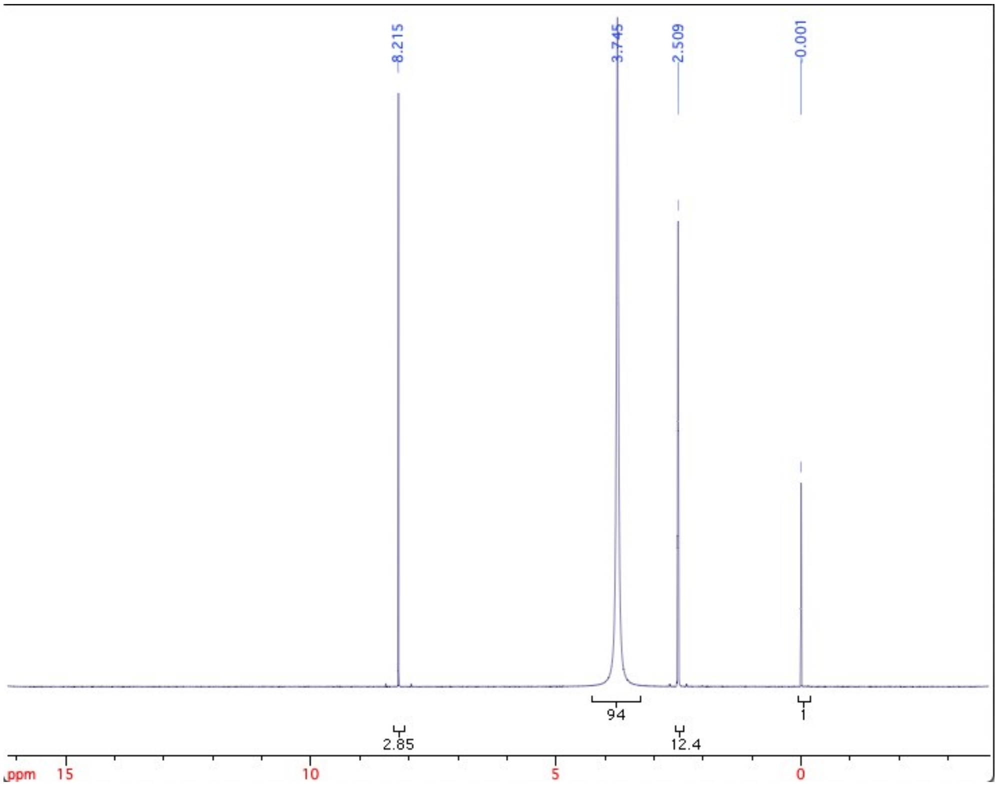

Figure S10: Formic Acid Degradation room temperature T = 5 h. Two trials.

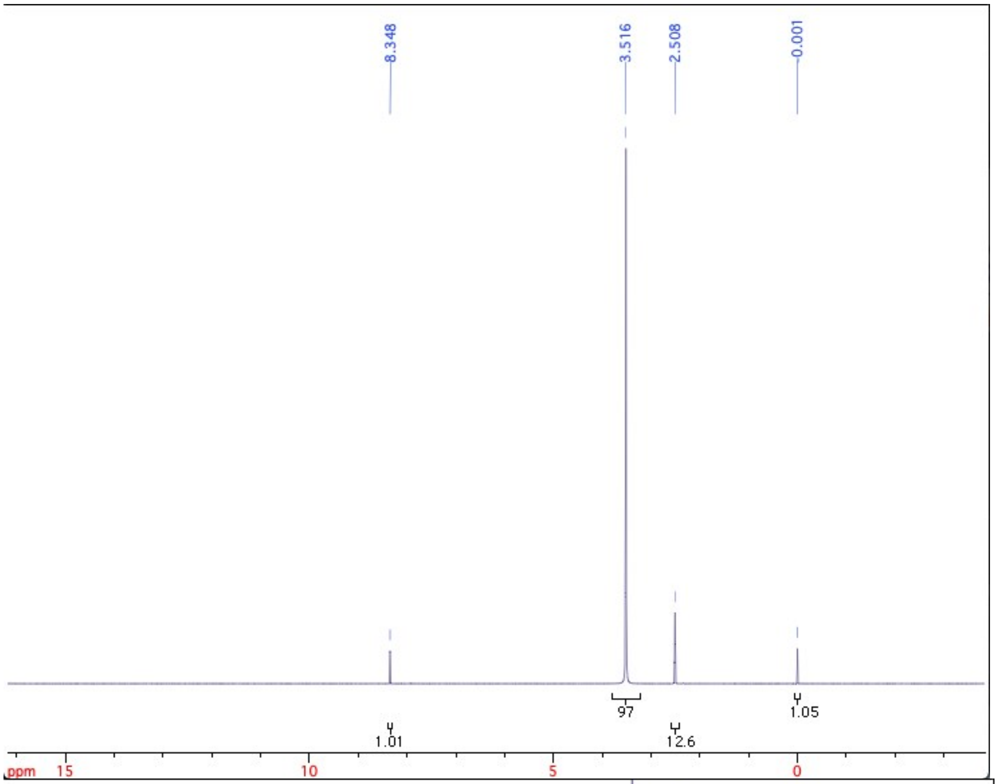

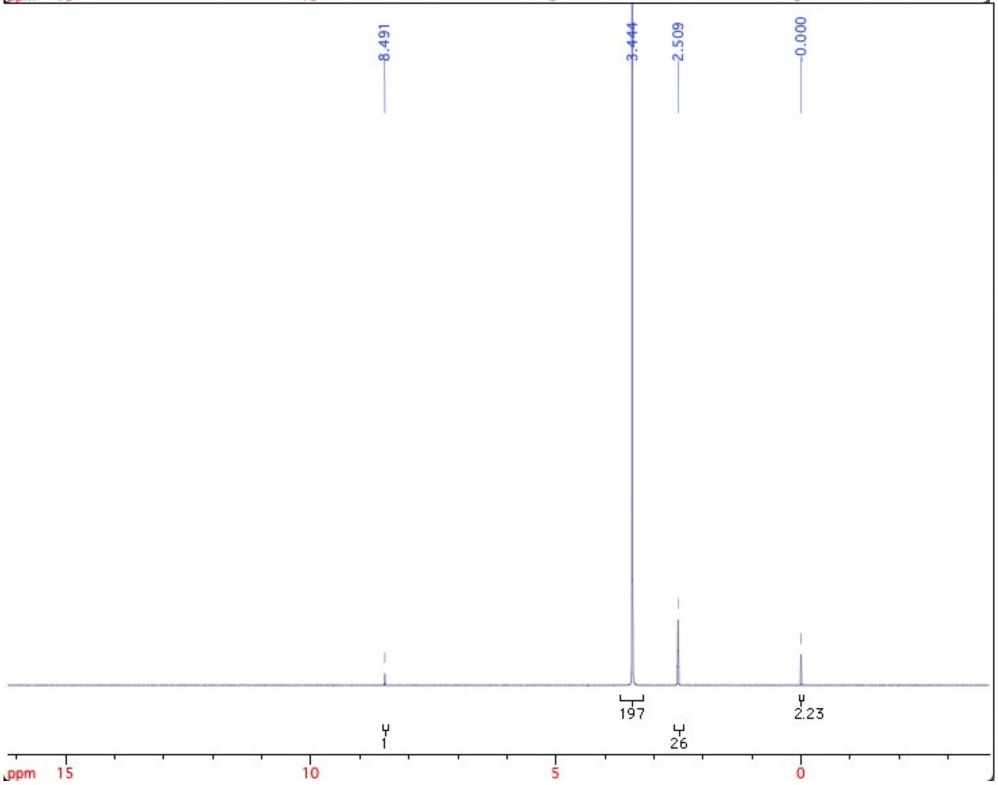

Figure S11: Formic Acid Degradation 50 °C T = 5 h. Two trials.

References:

(1) Navarro, M.; Smith, C. A.; Albrecht, M. Enhanced Catalytic Activity of Iridium(III) Complexes by Facile Modification of C,N-Bidentate Chelating Pyridylideneamide Ligands. *Inorg. Chem.* **2017**, *56* (19), 11688–11701. https://doi.org/10.1021/acs.inorgchem.7b01654.
